# Supplementary material for: Real-World Outcomes in FLT3-ITD Mutated Acute Myeloid Leukemia: Impact of NPM1 Mutations and Allogeneic Transplantation in a Retrospective Unicentric Cohort
Source: J Clin Med. 2025 Jul 18;14(14):5110. doi: 10.3390/jcm14145110 (PMC12294857; doi:10.3390/jcm14145110)
Supplement: Supplementary file 1 [file jcm-14-05110-s001.zip › jcm-3743895-supplementary.pdf]

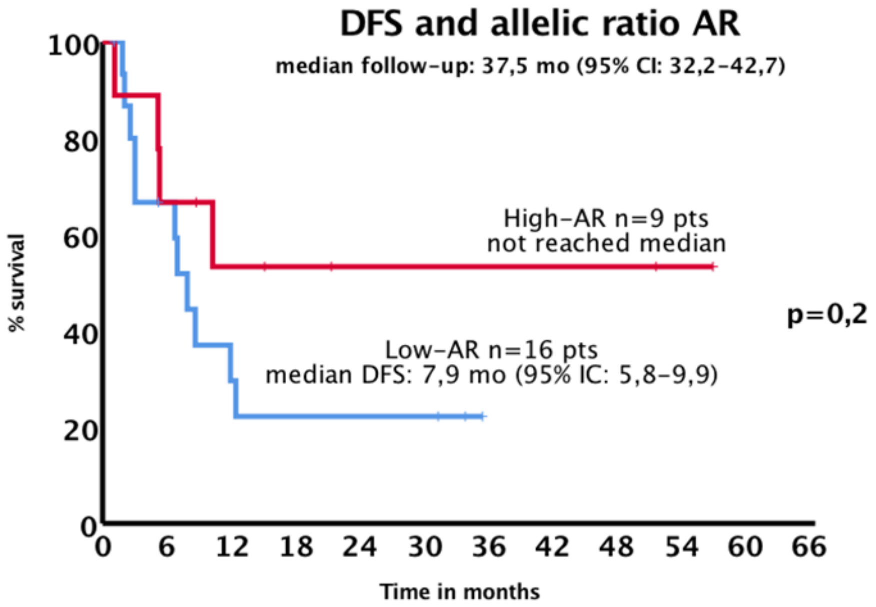

Supplementary Figure S1: "Disease free survival" related to allelic ratio groups. Log-rank test with Kaplan-Meier survival curves showed no significant statistical difference in disease free survival between High-AR group and low-AR group.

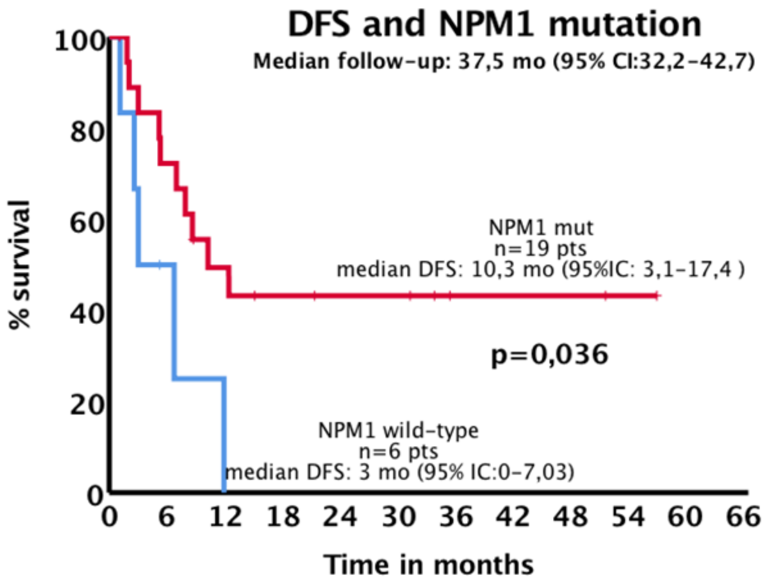

Supplementary Figure S2: "Disease free survival" related to *NPM1* mutation. Log-rank test with Kaplan-Meier survival curves showed a significant statistical difference in disease free survival between the group of patients with *NPM1* mutation and the group with wild-type *NPM1*

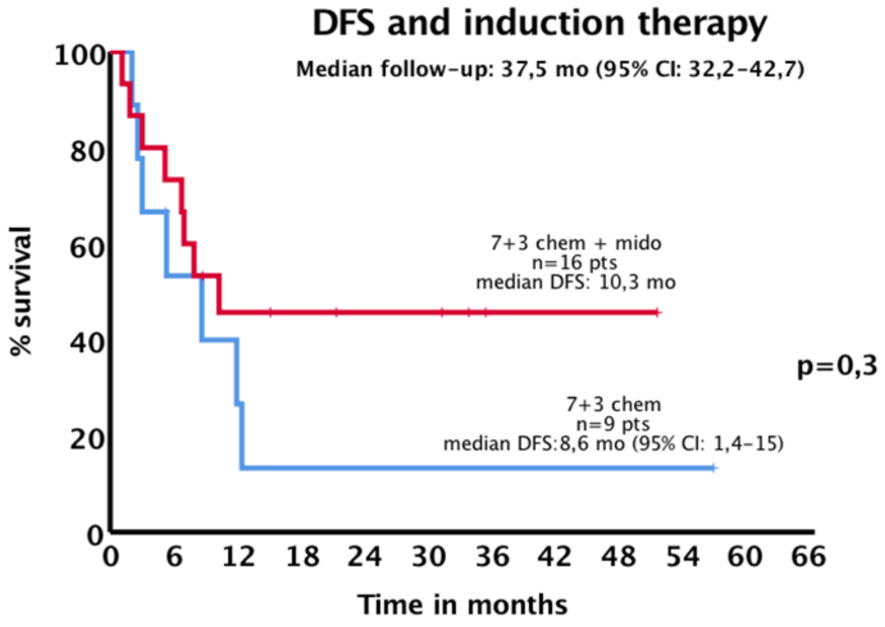

Supplementary Figure S3: "Disease free survival" related to induction therapy. Log-rank test with Kaplan-Meier survival curves showed no significant statistical difference in disease free survival between patients treated with 7+3+mido and patients no submitted to midostaurin during induction therapy

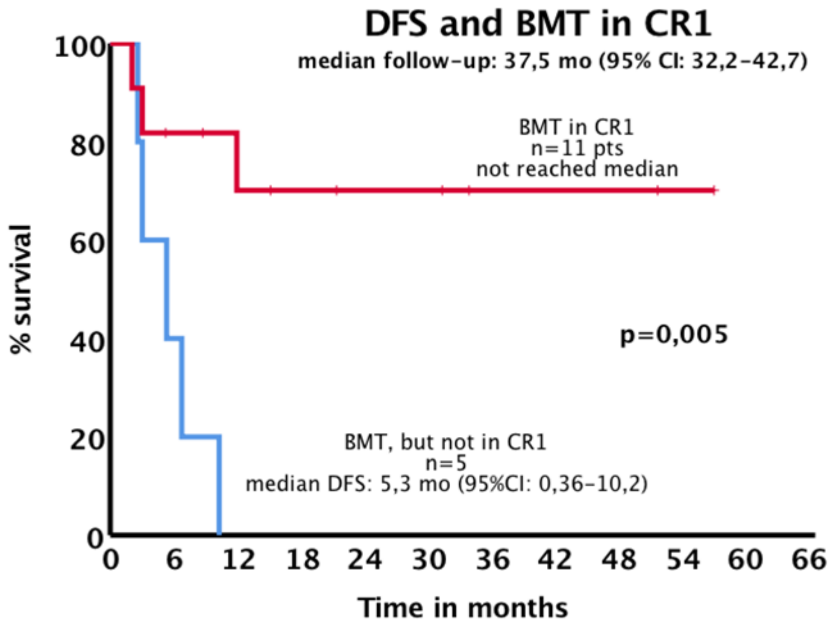

Supplementary Figure S4: "Disease free survival" related to bone marrow transplantation (BMT) in patients in remission after the first induction therapy (CR1). Log-rank test with Kaplan-Meier survival curves showed a significant statistical difference in disease free survival between patients treated with BMT and transplant in CR1 and patients not submitted to transplantation in CR

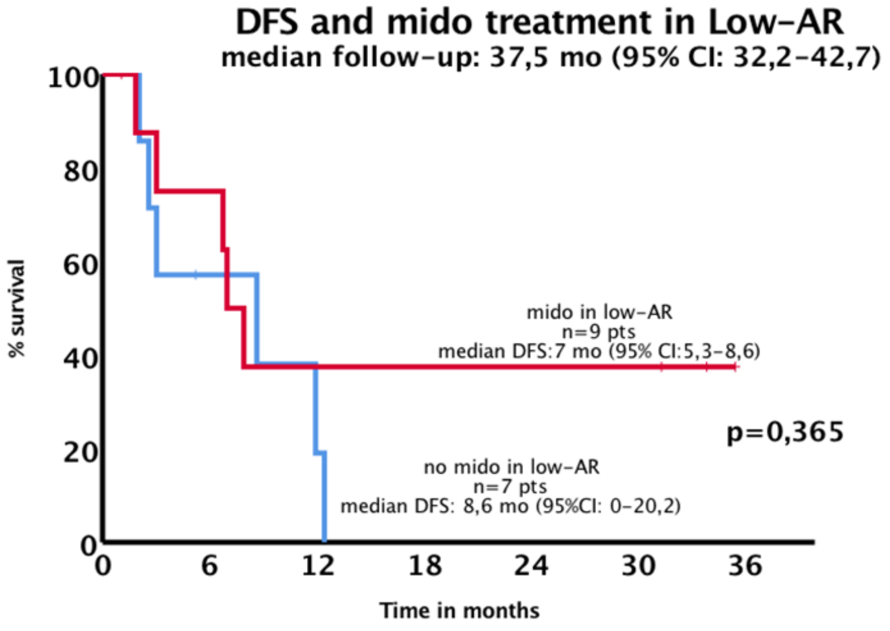

Supplementary Figure S5: "Disease free survival" related to induction therapy with midostaurin in low-AR group. Log-rank test with Kaplan-Meier survival curves showed no significant statistical difference in disease free survival between midostaurin and no midostaurin treated patients in low-AR group.

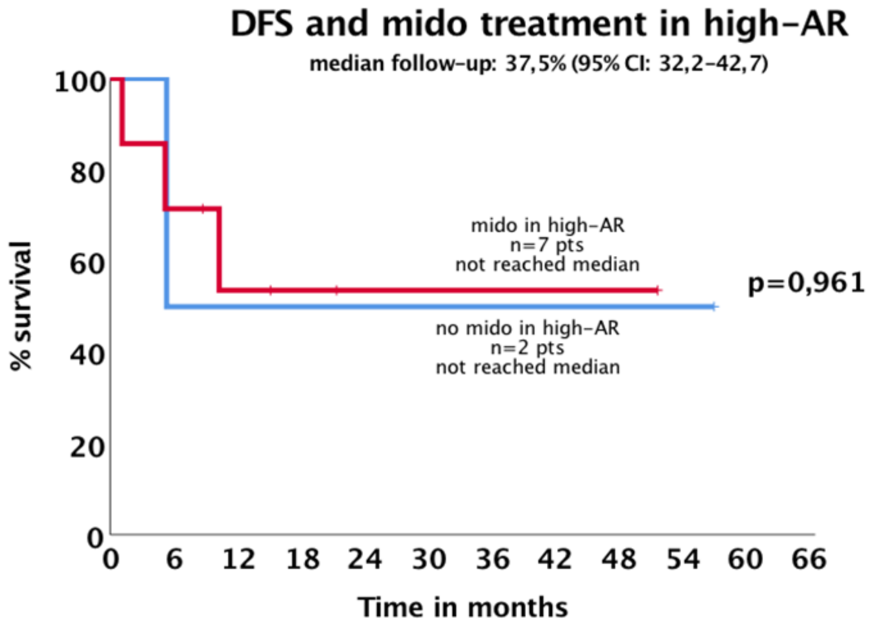

Supplementary Figure S6 "Disease free survival" related to induction therapy with midostaurin in high-AR group. Log-rank test with Kaplan-Meier survival curves showed no significant statistical difference in disease-free survival in high-AR group when treated with or without midostaurin.

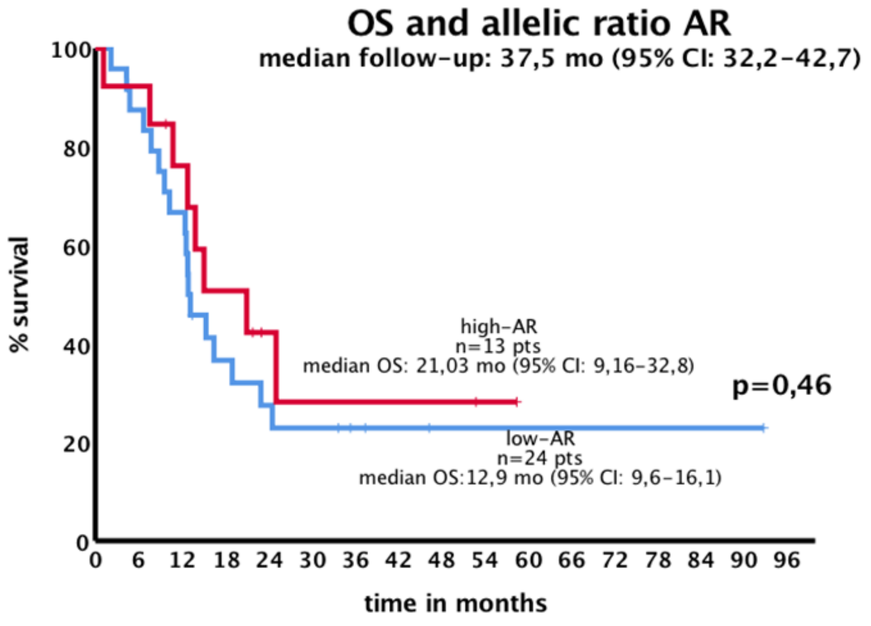

Supplementary Figure S7 "Overall survival" related to allelic ratio groups. Log-rank test with Kaplan-Meier survival curves showed no significant statistical difference in overall survival between high-AR group and low-AR group.

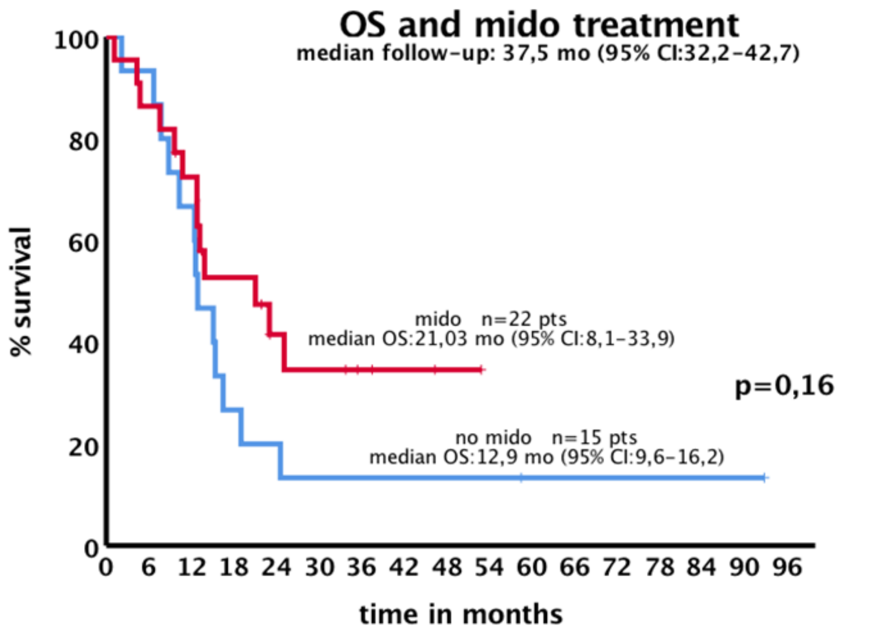

Supplementary Figure S8 "Overall survival"related to induction therapy with or without midostaurin. Log-rank test with Kaplan-Meier survival curves showed no significant statistical difference in overall survival between patients treated with 7+3+mido and patients no submitted to midostaurin during induction therapy.

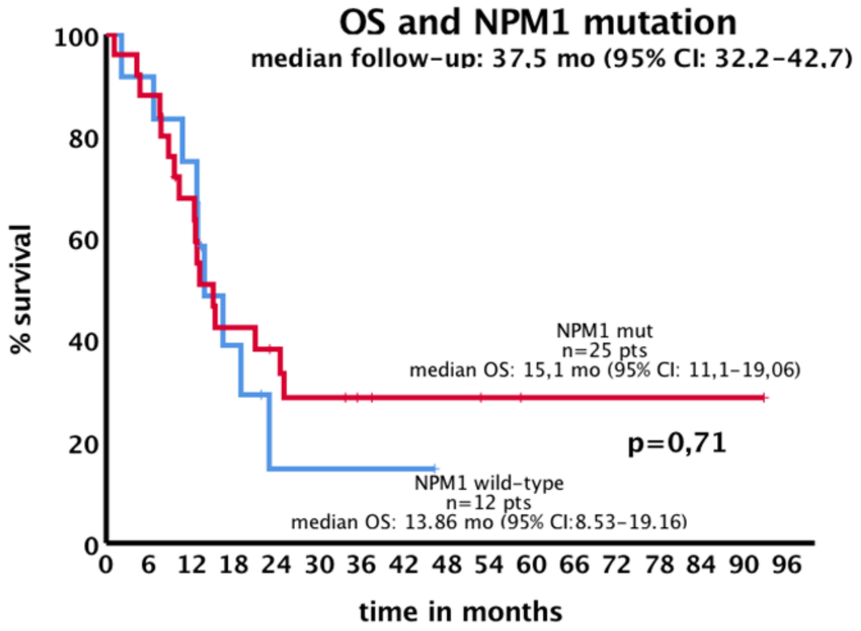

Supplementary Figure S9: "Overall survival" related to *NPM1* mutation. Log-rank test with Kaplan-Meier survival curves showed no significant statistical difference in overall survival between the group of patients with *NPM1* mutation and the group with wild-type *NPM1*.

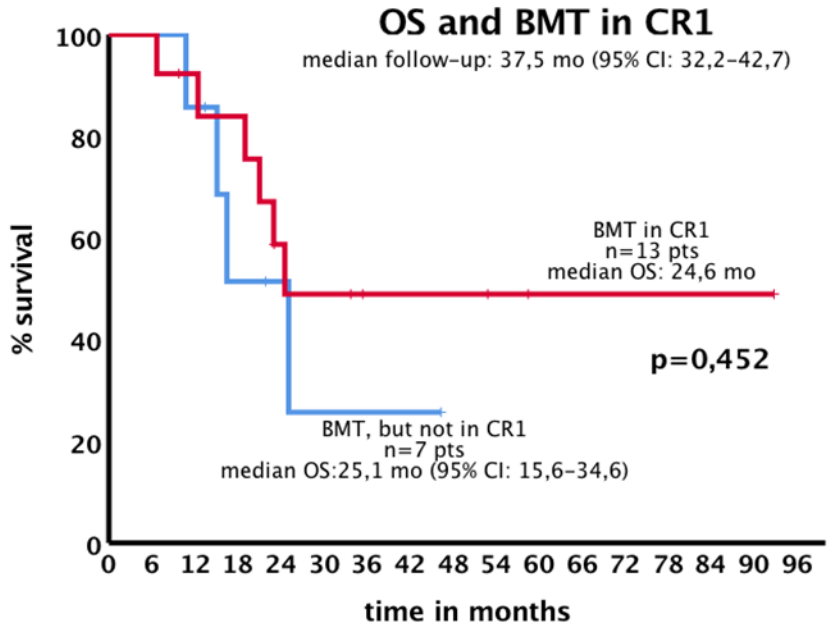

Supplementary Figure S10: "Overall survival" related to bone marrow transplantation (BMT) in patients in remission after the first induction therapy (CR1). Log-rank test with Kaplan-Meier survival curves showed no significant statistical difference in overall survival between patients treated with BMT and transplant in CR1 and patients not submitted to transplantation in CR

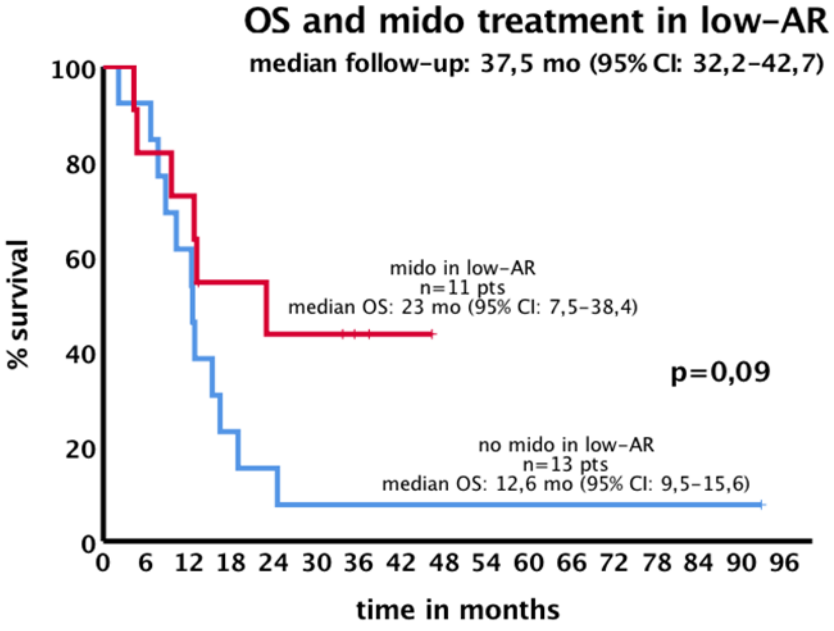

Supplementary Figure S11: "Overall survival" related to induction therapy with midostaurin in low-AR group. Log-rank test with Kaplan-Meier survival curves showed no significant statistical difference in overall survival between midostaurin and no midostaurin treated patients in low-AR group.

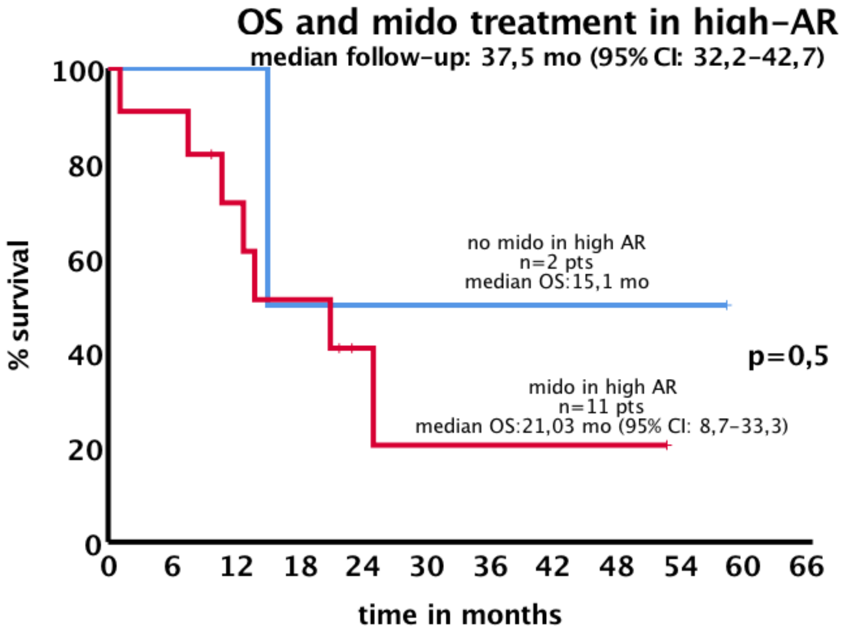

Supplementary Figure S12: "Overall survival" related to induction therapy with midostaurin in high-AR group. Log-rank test with Kaplan-Meier survival curves showed no significant statistical difference in overall survival between midostaurin and no midostaurin treated patients in high-AR group.
